# The effect of expertise, target usefulness and image structure on visual search

**DOI:** 10.1186/s41235-021-00282-5

**Published:** 2021-03-12

**Authors:** Samuel G. Robson, Jason M. Tangen, Rachel A. Searston

**Affiliations:** 1 School of Psychology, The University of Queensland, St Lucia, 4072 QLD Australia; 2 School of Psychology, The University of Adelaide, Adelaide, 5005 SA Australia

**Keywords:** Attention, Domain-specificity, Fingerprints, Perceptual expertise, Visual search

## Abstract

Experts outperform novices on many cognitive and perceptual tasks. Extensive training has tuned experts to the most relevant information in their specific domain, allowing them to make decisions quickly and accurately. We compared a group of fingerprint examiners to a group of novices on their ability to search for information in fingerprints across two experiments—one where participants searched for target features within a single fingerprint and another where they searched for points of difference between two fingerprints. In both experiments, we also varied how useful the target feature was and whether participants searched for these targets in a typical fingerprint or one that had been scrambled. Experts more efficiently located targets when searching for them in intact but not scrambled fingerprints. In Experiment 1, we also found that experts more efficiently located target features classified as more useful compared to novices, but this expert-novice difference was not present when the target feature was classified as less useful. The usefulness of the target may therefore have influenced the search strategies that participants used, and the visual search advantages that experts display appear to depend on their vast experience with visual regularity in fingerprints. These results align with a domain-specific account of expertise and suggest that perceptual training ought to involve learning to attend to task-critical features.

## Significance statement

In the forensic sciences, decisions about the source of a handwriting sample, cartridge case, shoe print or fingerprint rest on the perceptual judgments of human examiners. These decisions are often highly consequential, yet we lack a comprehensive scientific understanding of the nature and acquisition of perceptual expertise in these domains. Criticisms about the reliability and validity of the forensic sciences have prompted efforts to understand the nature of forensic pattern matching expertise, including in fingerprint examination. Fingerprint examiners can accurately distinguish fingerprints originating from the same source and those originating from different sources, but what cognitive mechanisms underlie this ability? In this study, we use fingerprint examination as a case domain to investigate how well perceptual experts can search for features in domain-relevant tasks and how experts differ from novices. We found that examiners search efficiently for useful fingerprint features, but not less useful features, nor features in scrambled fingerprint images. It may follow that judgments made by perceptual experts are valid only in the domain with which they have had considerable practice. Effective training may also rely on developing a sensitivity to the most useful features in one’s particular domain.

## Background

Many experts possess extraordinary perceptual and cognitive abilities for stimuli specific to their domain of practice. Medical diagnosticians, for example, can rapidly detect whether a chest radiograph contains an anomaly (Carrigan et al., 2019; Kundel & Nodine, 1975) or whether a mammogram is abnormal (Brennan et al., 2018; Carrigan et al., 2018; Evans et al., 2013). Feats of expertise like these exist in many fields (e.g., Abernethy, 1991; Gobet & Simon, 1996; Thompson & Tangen, 2014). To accurately and consistently determine the most appropriate response in tasks of this sort requires an ability to detect, locate and evaluate stimulus features (Seitz, 2020). A sensitivity to useful features may help to explain why experts perform so well on these tasks relative to the general population. In this study, we use visual search tasks to explore whether experts are more sensitive than novices to useful features (as opposed to less useful features) and whether any such advantage extends to stimuli that are unrelated to the experts’ specific domain.

Over many hours of training, individuals accumulate an extensive visual memory for category instances. In doing so, they typically develop an implicit appreciation for how visual features tend to co-occur and how cues or patterns relate to different response options (Brooks, 2005; Brunswik, 1955; Chase & Ericsson, 1982; Klein, 2008; Tanaka et al., 2005; Wiggins et al., 2014). Individuals can also learn to use cue-based associations from memory to make decisions quickly and accurately—a skill known as *cue utilization* (Lansdale et al., 2010; Sturman et al., 2019). For instance, the location of two objects on a screen and their relative trajectory may signal to an air-traffic controller that a change of course is needed (Loft et al., 2007). When using cues effectively, viewers rapidly attend to a small number of highly relevant features to arrive at accurate decisions, which has the benefit of reducing cognitive load (Brouwers et al., 2016; Carrigan et al., 2020; Curby & Gauthier, 2007; Curby et al., 2009; Sturman et al., 2019). With this skill, experts tend to become sensitive to the most useful features of a domain and diverge from novices in what they perceive as salient (e.g., Carrigan et al., 2019). Eye-tracking studies, for example, have demonstrated that medical diagnosticians are quicker to fixate on abnormalities in medical images than novices, and spend more time dwelling on anomalous regions (Krupinski, 1996; Krupinski et al., 2013; Kundel et al., 1978; van der Gijp et al., 2017). Similar expert-novice differences in attention exist in several other applied domains (Mann et al., 2007; Maturi & Sheridan, 2020; Reingold et al., 2001; Sheridan & Reingold, 2014; Ziv, 2016). But do the visual advantages that experts possess extend beyond their domain of formal training?

Visual search comprises two stages, the first of which is nonselective, global or holistic, and occurs when a viewer extracts the image statistics or ‘gist’ of a scene (Drew et al., 2013; Hoffman, 1979; Kundel et al., 2007; Swensson, 1980; Torralba et al., 2006; Wolfe, 1994; Wolfe et al., 2011). During this initial stage, a viewer gleans the global context of the scene or image where a target is located, and this context guides attention to regions of interest. Attention can be involuntarily drawn to salient colors and shapes, but also guided based on the expectations and experience of the viewer (Boot et al., 2009; Itti & Koch, 2001; Wolfe, 2020; Wolfe & Horowitz, 2017). For example, if searching for a pen, our attention will be drawn toward a horizontal surface like a desk because our experiences with objects and scenes of this sort indicate that it is the likely place for such an object to be located (Wolfe et al., 2011).

Similarly, an expert’s refined cognitive representation for the statistical regularities of their domain—how features tend to appear and co-occur—will guide them to particular locations (Chun & Jiang, 1998; Jiang et al., 2013; Torralba et al., 2006; Wolfe et al., 2011). A quick glance at a medical image, for instance, will cue a radiologist toward a suspicious mass because the anatomical context sets up location priors about where such an abnormality is likely to be (Carrigan et al., 2019). The context of a target is therefore critical to how attention is guided and to an expert’s sensitivity to useful features. Disrupting this global structure may then inhibit an expert’s performance.

Many perceptual and cognitive advantages that experts possess are in fact domain-specific (Carrigan et al., 2019; Chase & Simon, 1973a, b; Curby et al., 2009; Diamond & Carey, 1986, McKeeff et al., 2010; Memmert et al., 2009; Nodine & Krupinski, 1998; Richler et al., 2011; Sims & Mayer, 2002). Expert chess players, for example, demonstrate better recall than novices for briefly presented arrangements of chess pieces, but only when the pieces are configured in a typical game-like manner (Chase & Simon, 1973a, b; de Groot, 1965). When these pieces are randomized, an expert’s recall advantage disappears. Chess masters also attend to the most critical pieces and positions on a chess board when typical game scenarios are presented, but not when the pieces are placed in random positions (Bilalić et al., 2012; Reingold et al., 2001; Sheridan & Reingold, 2014). The implication of this research is that chess masters can mentally group pieces as ‘chunks’ because they have developed a refined cognitive representation for how chess pieces tend to appear and cluster together (Chase & Simon, 1973a, b; Gobet & Simon, 1996). Chess masters can therefore encode, store and retrieve large amounts of information by memorizing it in a way that is coherent and familiar. When the typical chess board configuration is scrambled, on the other hand, a fine-tuned appreciation for patterns of chess pieces cannot aid recall because the way the board is arranged no longer bears any resemblance. Many perceptual and cognitive skills that experts have are highly dependent on domain-specific knowledge of visual structure in much the same way.

There are, however, skills that appear to generalize beyond narrow fields of formal training (see Cain et al., 2012; Carrigan et al., 2020; Green & Bavelier, 2003, 2006; Sowden et al., 2000; Sunday et al., 2018). Perhaps experts in some fields are selected for because they possess above average general perceptual abilities, or perhaps their training has granted them skills that apply to a broad range of stimuli and tasks. In fact, a measure of domain general visual expertise—the Novel Object Memory Test (NOMT)—has been designed and validated in several studies (Sunday et al., 2018; Carrigan et al., 2020). It is unclear whether a sensitivity to useful features, and the ability to search for them, is domain-specific or driven by more general pattern recognition capacities. In the present study, we use fingerprint examination as case domain to examine whether experts are more sensitive to useful features than novices and whether this sensitivity is domain-specific or domain general. Below we describe this field before outlining our experiments and hypotheses.

### Fingerprint examination

Fingerprint examiners spend significant portions of their working day looking at and comparing fingerprints to determine whether they match. Many of these examiners work in this field for many years. For example, the average experience among 36 examiners reported by Tangen et al. (2020) was 16.4 years. Fingerprint examiners in Australia—where we conduct most of our research—undergo five years of training. To be accredited as an examiner by the Australian Forensic Science Assessment Body (AFSAB), they must then pass a final multi-day test of their abilities. Accredited examiners also apply for recertification every five years where they receive further assessments of their competency (AFSAB, 2019).

Examining and comparing highly structured fingerprint impressions for several hours every day suggests that these examiners ought to be able to determine whether two fingerprints match. Several experiments demonstrate that these examiners are more accurate than chance and novice controls at discriminating whether two prints match (e.g., Tangen et al., 2011; Thompson et al., 2013). Examiners also possess many other non-analytic, fingerprint-related abilities (e.g., matching different fingerprints from the same hand based on ‘style’ or matching prints that are heavily clouded in noise; Searston & Tangen, 2017b; Thompson & Tangen, 2014). In collaborating with these examiners for several years, it became apparent that one under-explored aspect of a fingerprint examiner’s expertise is their visual search ability. For example, the traditional method for identifying fingerprints has been described as a careful comparison of fingerprint features in two different fingerprint impressions (Ashbaugh, 1999); examiners are typically said to select a small cluster of features in one print and search for this cluster, or any deviations from it, in a comparison print.

In the present study, we investigate the visual search skills of fingerprint examiners across two tasks. The first task assesses the ability to spot corresponding features (Experiment 1), and the second assesses the ability to spot differences (Experiment 2). Like other experts, fingerprint examiners appear sensitive to the more useful features within fingerprints; examiners and novices often consider different kinds of features to be useful (Robson et al., 2020), and examiners, more so than novices, tend to constrain their attention to fewer but more distinctive regions (Roads et al., 2016). In these two experiments, we will examine whether professional examiners are more sensitive to useful fingerprint information, in which case they ought to be more efficient than novices at locating useful features and changes (but not less useful features/changes). We will also examine whether any expert advantage in searching for useful features is limited to fingerprints or whether it persists even when the fingerprints in the task are scrambled. Vogelsang et al. (2017) have demonstrated that examiners process upright fingerprints very efficiently, but not so when the prints are inverted. Similarly, if a sensitivity to useful features is domain-specific, then scrambling a fingerprint may severely diminish an examiner’s ability to locate information because the structure of the print can no longer efficiently guide their search.

## Experiment 1

In Experiment 1, we designed a ‘Find-the-Fragment’ task to investigate whether expert fingerprint examiners are more efficient than novices at locating features within prints. In their work, examiners routinely select a small cluster of features in one print and search for this group within the vast array of ridge detail in a comparison print. Such a task resembles the classic visual search paradigms where participants locate a target (e.g., a green X) among a jumble of other shapes and colors (Treisman & Gelade, 1980). Similarly, the Find-the-Fragment task that we use in Experiment 1 instructs participants to locate a small ‘fragment’ of ridge detail within a larger fingerprint image as quickly as possible.

Tasks of this sort have been used in previous studies of visual search in applied domains. For example, Maturi and Sheridan (2020) used eye-tracking methods to compare how well expert and novice musicians could match a small section of musical score to its corresponding counterpart in a larger music score. Expert musicians were more accurate, spent longer looking at relevant regions, and returned to the target feature less often than non-musicians. Hicklin et al. (2019) also created a similar visual search task with fingerprint materials. They presented a target template either without any visual context or within the context of a fingerprint and instructed participants to search for the target in a second print. Examiners more easily spotted target features when presented with context, but Hicklin and colleagues made no comparison between experts and novices in their study. Building on this work, we examine how expertise, target usefulness and image structure affect visual search. Specifically, we compare fingerprint examiners to novices on a fingerprint-like visual search task and manipulate both how useful the target features are and the structure of the image that participants search for these targets in.

As we have outlined, perceptual experts show signs that they are more sensitive to diagnostic cues within their domain, resulting in superior performance compared to novice participants. However, the superior capabilities of many experts diminish when the subject matter, such as a fingerprint, is changed or disrupted. An expert’s expectations about where useful features might be located within a fingerprint may no longer facilitate search if the spatial layout or context that typically cue attention to useful features are not present. We therefore hypothesize that examiners will outperform novices on the Find-the-Fragment task only when locating useful fragments within intact fingerprints. In essence, we predict a three-way interaction where the groups differ in the useful-intact version of the task, but not in any of the other conditions. Prior work comparing fingerprint examiners and novices reveals large expert-novice effect sizes (e.g., Tangen et al., 2011; Thompson & Tangen, 2014) and thus we expect the effect of expertise in this critical condition to be large as well.

## Method

### Preregistration

All methods, materials, event sequences, experimental code, sensitivity analyses, hypotheses and planned analyses were preregistered on the Open Science Framework prior to collecting data. The preregistered project can be found here: https://osf.io/azesx.

### Participants

*Experts* In total, we collected data from 44 fingerprint examiners who are accredited in their jurisdiction and court practicing. We originally planned to collect data from at least 30 participants, which provided sufficient power to (> 0.9) to detect a medium effect size for all planned analyses, but we intended to collect data from as many experts as possible. Every participant completed this experiment as well as Experiment 2 along with a suite of other tasks presented in a randomized order during the same testing session. These other tasks address different research questions beyond the scope of this paper.

*Novices* We collected data from 44 novice participants that were age (± 2 years), gender and education matched to each of the expert participants (see Table 1). We yoked each novice to their matched expert ‘twin’ such that they were shown identical event sequences and completed each task in the same order. These novices had no formal experience with fingerprint examination and were recruited from the University of Adelaide, The University of Queensland, and Murdoch University communities, and some from the general public. To recruit novices who were motivated to perform well, we compensated them with 20 Australian dollars per hour of their time. They were also given an additional $5 if they could reach or surpass the performance of their expert ‘twin’ in the task.

**Table 1:** Demographic details of the expert and novice participants

	Experts	Novices
Age	*M* = 43.6 (SD = 8.41, range 29–60)	*M* = 43.6, (SD = 8.69, range 30–62)
Reported gender	25 females, 19 males	25 females, 19 males
Education	3-year undergraduate degree (min)	3-year undergraduate degree (min)
Professional experience (years)	*M* = 14.9 (SD = 7.75, range 5–40)	*M* = 0 (SD = 0)

Each novice participant was age-matched, gender-matched and education-matched with an expert participant

### Design

**Fig. 1:**
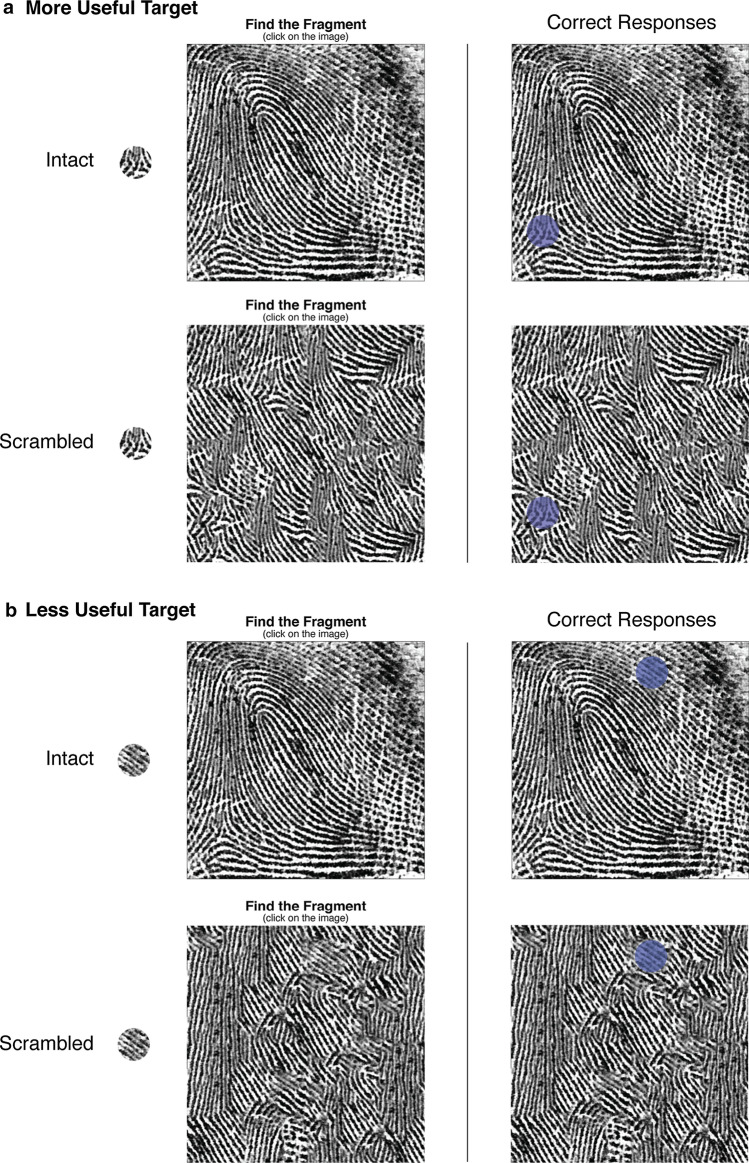
A schematic diagram of the Find-the-Fragment task used in Experiment 1. On the left, we provide an example of each of the trial types: the array fingerprint was either an intact fingerprint or scrambled, and the target feature was either a ‘more useful’ feature (**a**), or ‘less useful’ feature (**b**). When generating the scrambled fingerprints, we kept the location and appearance of the fragments identical to those in the intact prints. The correct locations of the fragments for each example are displayed on the right

We employed a 2 (Expertise: expert, novice; between subjects) × 2 (Target: more useful, less useful; within subjects) × 2 (Structure: intact, scrambled; within subjects) mixed design ‘yoked’ to expertise. Participants in each of the 48 trials saw a small fragment of fingerprint detail presented on the left of the screen and a larger fingerprint image (the array) on the right. They were instructed to click where they thought the smaller fragment was located in the larger array (see Fig. 1). Half of the trials instructed participants to locate ‘more useful’ target fragments and half required them to locate ‘less useful’ fragments. Half of the trials also instructed participants to find these target fragments within intact, unaltered fingerprint images and half required them to locate fragments within scrambled prints. We controlled for the target fragment’s location and appearance across the intact and scrambled conditions by keeping these unchanged when creating the scrambled images.

### Materials

*Intact array images* We used 72 of the 100 fingerprint images used in Robson et al.’s (2020) study that we considered to be clearest and least noisy. Of these fingerprints, 26 were latent prints—impressions left under uncontrolled conditions (to resemble crime-scene prints). The other 46 were tenprints—fingerprints collected under controlled conditions using ink and rolled from nail to nail. Each image had been cropped such that the entire image was filled with ridge detail (750 × 750 pixels).

*Fragment images* To generate the ‘more useful’ and ‘less useful’ target fragments, we used data collected in a previous study (Robson et al., 2020) where we asked experts and novices to mark a feature that they considered to be *most useful* if they were to distinguish the fingerprint from other prints, and also to mark a feature that they considered *least useful*. We observed that features classified as useful by fingerprint examiners tend to include aspects such as the core (i.e., the center of the fingerprint), the delta (i.e., a Y-shaped structure where two parallel ridges diverge, as depicted in Fig. 1) and other smaller discontinuities in a print, such as ‘ridge endings’ and ‘lakes’ (Robson et al., 2020). Features identified as less useful included unremarkable sections of ridge detail, smudges and areas lacking clarity. We used these spatial judgments as center points to generate thousands of circular fragments with various radii. In a pilot experiment (preregistered here: https://osf.io/3g4e7), we presented novices with a version of the Find-the-Fragment task where the target fragment gradually increased in size every five seconds. Participants were 50% correct when locating fragments that were 1.45% of the area of the larger fingerprint. We used this fragment size in the present study because 50% performance was equidistant from floor and ceiling, and therefore seemed appropriately difficult. We randomly sampled eight ‘more useful’ fragments and eight ‘less useful’ fragments of this size from each of the 72 fingerprints in this experiment, a total of 1152 possible combinations.

*Scrambled images* The purpose of the scramble was to remove the fingerprint-like structure of the images while retaining low-level visual properties and the location and appearance of the fragments. We generated 16 different scrambled versions of the 72 intact fingerprints (one for each of the randomly sampled fragments). We deselected the area where the fragment was located, deleted and replaced one quadrant of the print with alternative information using Photoshop’s Content Aware Fill tool.^1^ We continued this process for the other three quadrants moving around clockwise until the entire image was scrambled, except for the fragment itself. We began the procedure in each of the four quadrants in equal number. In total, we created a pool of 1152 scrambled prints.

### Measures

We measured performance on the Find-the-Fragment task using a speed–accuracy measure known as the Balanced Integration Score (BIS; Liesefeld et al., 2015). BIS was devised to give equal weighting to both response time and accuracy. It is calculated by standardizing response time and accuracy for only the correct trials (incorrect trials are affected more by speed–accuracy trade-offs) to bring both to the same scale. One standardized score is then subtracted from the other to provide each participant with a single speed–accuracy score for each condition. BIS is considered a robust measure of speed–accuracy because it is insensitive to individual differences in speed–accuracy trade-offs (Liesefeld & Janczyk, 2019). The average BIS in a dataset is zero and the standard deviation is one. Positive scores indicate performance that is above the mean and negative scores indicate scores that fall below the mean.

In our preregistration, we planned to compare experts and novices on the proportion of correct responses (accuracy) and their Rate Correct Score (RCS), which measures speed–accuracy as the proportion of correct responses per second (Woltz & Was, 2006). However, we have since discovered that the accuracy data did not adequately reflect performance, and RCS, while easy to interpret, has many limitations (see Liesefeld & Janczyk, 2019; Vandierendonck, 2018). Nonetheless, data analytic scripts for each measure can be found here: https://osf.io/94zf6.

### Software

The video instructions and the task were presented to participants on a 13-inch MacBook Pro or MacBook Air laptop screen, with over-ear headphones. The software used to generate the trial sequences, present stimuli to participants and record their responses, was developed in the open source ‘Community Edition’ of LiveCode (Version 9.5.1).

### Procedure

Participants first read information about the project and watched an instructional video about the task with examples. They were then presented with their 48 randomly sequenced Find-the-Fragment trials. After clicking the array, immediate feedback was provided. If participants made an incorrect click (they did not click where the fragment was located) they were presented with a red cross, a blue semitransparent circle highlighting the correct location (as shown in Fig. 1), and they heard a dull tone. However, if a participant correctly clicked on the fragment in the array, they saw a green tick, a blue semitransparent circle highlighting the correct location, and heard a bright tone. There was a 1500-ms window for the feedback to appear and then another 500-ms blank screen before the images on the next trial appeared. All images remained on the screen until the participant made a response, but a text prompt appeared during the inter-trial interval if participants took longer than 15 s to respond, with the message: 'Try to respond in less than 15 seconds.' A progress bar in the bottom right-hand corner of the screen was displayed to indicate how many trials had been completed and how many remained.

## Results

**Fig. 2:**
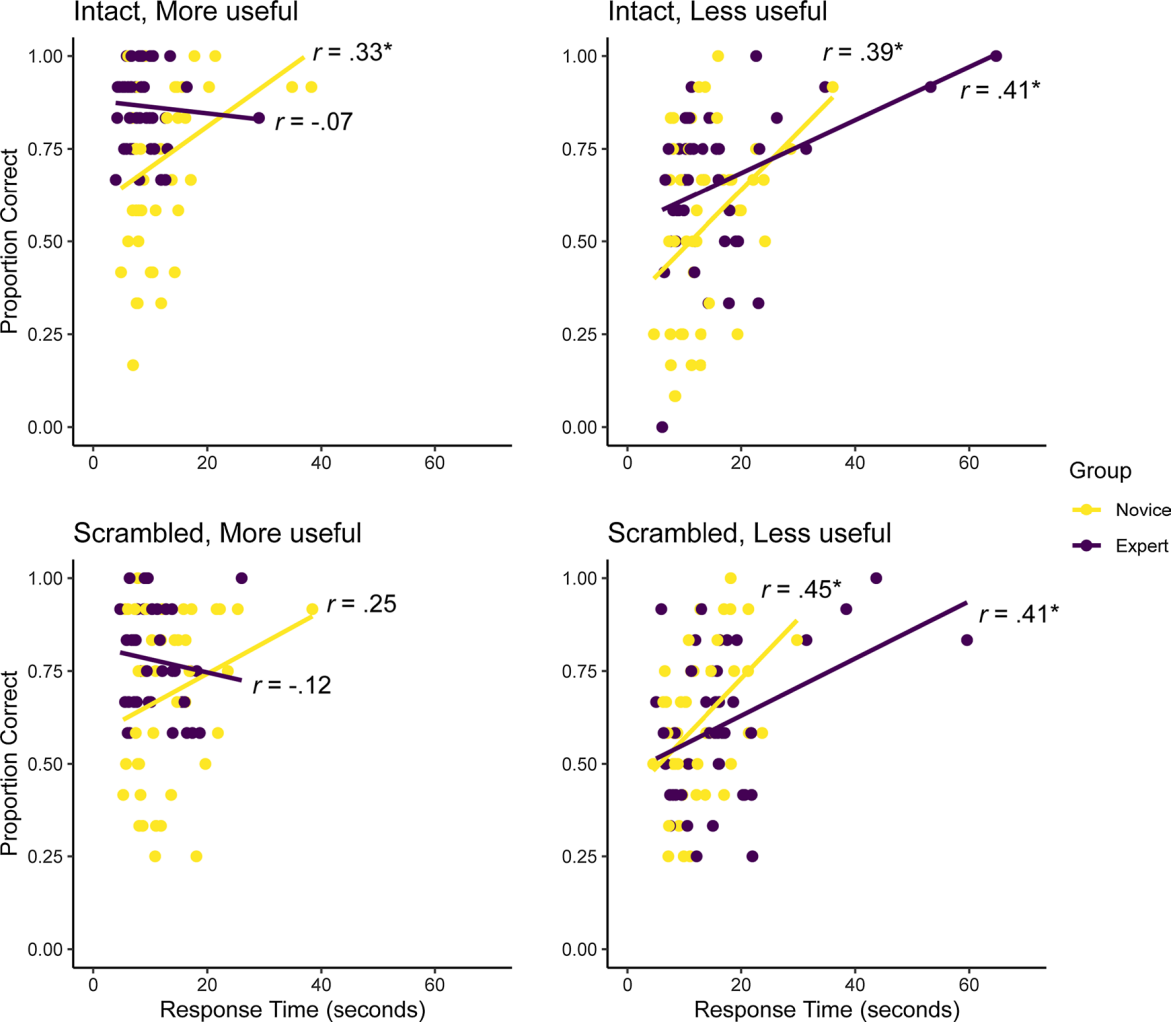
The correlations between speed and accuracy for each group across the four conditions in the Find-the-Fragment task. An asterisk (*) signifies a significant correlation (*p* < .05)

In this experiment, accuracy data were not homogenous nor normally distributed in every cell. Because the target fragment was present on every trial and participants could inspect the images for as long as they desired, there was a ceiling effect in accuracy. Overall, the time spent making a decision was moderately correlated with accuracy, *r*(86) = 0.53, *p* < 0.001. This correlation was similar for within experts, *r*(42) = 0.57, *p* < 0.001, and within novices, *r*(42) = 0.59, *p* < 0.001. We present the speed–accuracy correlations for each condition in Fig. 2. Some participants clearly prioritized accuracy while others prioritized speed, so speed–accuracy is a more suitable measure of task performance.

### Speed–accuracy (BIS)

**Fig. 3:**
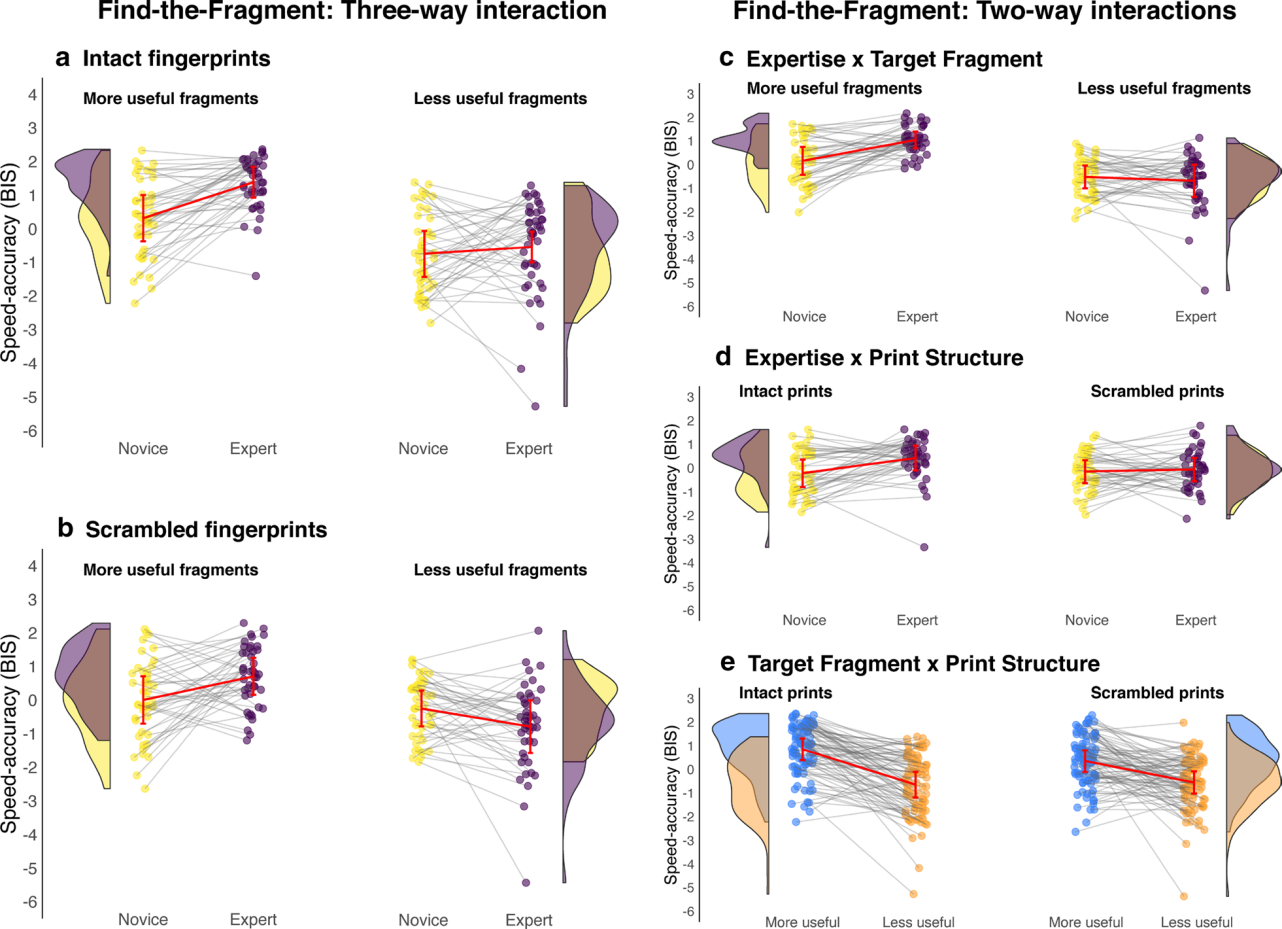
A visualization of the interactions for the speed–accuracy data (measured using Balanced Integration Scores) in the Find-the-Fragment task. We present the three-way interaction (left) comparing experts and novices for each fragment type for the intact trials (**a**) and scrambled trials (**b**). We also present all three two-way interactions (**c**–**e**). In all but panel **e**, the expert data are colored purple and the novice data yellow. Each participant’s individual performance is represented by the small circles, with grey lines connecting every expert to their yoked novice ‘twin’. The distributions depict the overlap in each group’s performance, the red error bars represent the confidence interval around each cell mean and a red line connects the group means for each cell. Panel E depicts the final two-way interaction comparing performance at finding more useful features (blue) and less useful features (orange) with each participant’s data connected by a grey line

The speed–accuracy data are presented in Fig. 3. We performed both a parametric analysis (three-way mixed factorial ANOVA) and nonparametric analysis (three-way Aligned Rank Transform [ART] ANOVA; Wobbrock et al., 2011) to analyze the BIS data because some cells were skewed or had heterogenous variances. The ART factorial approach aligns the data before applying averaged ranks so that common ANOVA procedures can be used. We found the pattern of effects in both models to be equivalent, so we only report the parametric ANOVA here.

The 2 (Expertise) × 2 (Target) × 2 (Structure) mixed methods ANOVA did not reveal a significant three-way interaction, *F*(1, 86) = 0.94, *p* = 0.334. However, the Expertise × Target interaction was significant, *F*(1, 86) = 21.61, *p* < 0.001, *η*^2^_G_ = 0.055. Experts (*M* = 1.04, SD = 0.9) were better than novices (*M* = 0.16, SD = 1.15) at locating the more useful fragments, *F*(1, 174) = 32.49, *p* < 0.001, *η*^2^_G_ = 0.157, but there was no difference between the experts (*M* = − 0. 68, SD = 1.33) and novices (*M* = − 0.52, SD = 1.04) for the less useful target fragments, *F*(1, 174) = 0.82, *p* = 0.367.

We also found a significant Expertise × Structure interaction, *F*(1, 86) = 8.06, *p* = 0.006, *η*^2^_G_ = 0.016. Experts (*M* = 0.42, SD = 1.48) were better at locating fragments in intact prints than novices were (*M* = − 0.22, SD = 1.26), *F*(1, 174) = 9.47, *p* = 0.002, *η*^2^_G_ = 0.052. However, the novices (*M* = − 0.15, SD = 1.02) performed as well as the experts (*M* = − 0.06, SD = 1.33) at locating target fragments in scrambled prints, *F*(1, 174) = 0.23, *p* = 0.633.

The Target × Structure interaction was also significant*, F*(1, 86) = 9.34, *p* = 0.003, *η*^2^_G_ = 0.018. Participants were better at locating the more useful target fragments (*M* = 0.85, SD = 1.02) than the less useful fragments (*M* = − 0.65, SD = 1.28) during the intact trials, *F*(1, 87) = 95.95, *p* < 0.001, *η*^2^_G_ = 0.285. Experts were also better at spotting the more useful target fragments (*M* = 0.35, SD = 1.09) than the less useful fragments (*M* = − 0.56, SD = 1.11) during the scrambled trials, *F*(1, 87) = 30.96, *p* < 0.001, but the effect size was smaller in comparison (*η*^2^_G_ = 0.147).

### Exploratory analyses

*Simple interactions* We predicted that experts would perform better than novices solely when finding useful fragments within intact fingerprints, but we did not find the expected three-way interaction that follows from this. We explored the results further by observing the simple interaction effects. As expected, there was an Expertise × Target interaction when observing solely the intact trials, *F*(1, 86) = 8.65, *p* = 0.004, *η*^2^_G_ = 0.036. Experts (*M* = 1.38, SD = 0.75) were better than novices (*M* = 0.32, SD = 1.13) at locating more useful target fragments in the intact prints, *F*(1, 86) = 27.23, *p* < 0.001, *η*^2^_G_ = 0.240. However, experts (*M* = − 0.54, SD = 1.40) performed similarly to novices (*M* = − 0.75, SD = 1.16) when locating less useful fragments in the intact prints, *F*(1, 86) = 0.57, *p* = 0.454. Unexpectedly, there was an Expertise × Target interaction for the scrambled trials as well, *F*(1, 86) = 16.95, *p* < 0.001. Counter to our predictions, experts (*M* = 0.70, SD = 0.90) were better than novices (*M* = 0.00, SD = 1.16) at locating the more useful fragments in scrambled prints, *F*(1, 86) = 10.05, *p* = 0.002, *η*^2^_G_ = 0.105. Moreover, the novices (*M* = − 0.29, SD = 0.86) outperformed the experts (*M* = − 0.82, SD = 1.16) at locating less useful target fragments in scrambled prints, *F*(1, 86) = 5.33, *p* = 0.023, *η*^2^_G_ = 0.058.

*Correlating performance and experience* When correlating years of experience with performance on the Find-the-Fragment task, we found no significant associations. Among experts, there was no correlation between their years of experience in fingerprint examination and their overall accuracy, *r*(42) = 0.05, *p* = 0.739, nor between years of experience and speed–accuracy, *r*(42) = − 0.19, *p* = 0.223. There was also no correlation between years of experience and accuracy when observing solely the useful-intact data, *r*(42) = − 0.14, *p* = 0.370, nor between years of experience and speed–accuracy for this cell, *r*(42) = 0.05, *p* = 0.755.

## Discussion

In Experiment 1, we compared the performance of professional fingerprint examiners to novices on a fingerprint-like visual search task. Our aim was to investigate whether any expert-novice differences in visual search depend on the usefulness of the target and the structure of the array image. We expected experts to outperform novices solely when locating useful target fragments in intact fingerprints. We did not find the expected three-way interaction, but the findings nonetheless partially support our hypothesis. Overall, experts were better than novices at locating the more useful target fragments, but they performed like novices when locating the less useful fragments. The examiners therefore appear highly sensitive to useful features, as has been demonstrated in many other domains of expertise (Carrigan et al., 2019; Krupinski, 1996; Krupinski et al., 2013; Mann et al., 2007; Maturi & Sheridan, 2020; Reingold et al., 2001; van der Gijp et al., 2017; Ziv, 2016). The vast amount of experience that examiners have accumulated over many years comparing highly structured prints has likely tuned them to the cues and features that are of greatest relevance for identifying fingerprints.

The fingerprint examiners also outperformed novices at locating fragments in intact fingerprints, but the two groups performed similarly when locating fragments in scrambled fingerprints. Our findings therefore align with other studies that have shown similar domain-specific expert performance (e.g., Carrigan et al., 2019; Chase & Simon, 1973a, b; Curby et al., 2009; Diamond & Carey, 1986, 2009; Sims & Mayer, 2002). It seems that an expert’s appreciation for visual regularity—or the context of a feature in an image—cues them to that target (Carrigan et al. 2019; Wolfe et al., 2011). When a fingerprint is scrambled, an expert’s familiarity with the typical spatial layout of a fingerprint can no longer guide their search as efficiently as it would when faced with a normal print. Participants, in general, also found it easier to locate the more useful fragments compared to the less useful fragments, but the difference was greater on the intact trials compared to the scrambled trials. Scrambled prints may contain more discontinuities than intact prints because of the way they were generated, and perhaps salient features are easier to locate in their typical context.

We did not find the three-way interaction that we expected because experts performed better than novices at searching for useful fragments not only in intact prints, but in scrambled prints as well. Examiners may have performed relatively well even in this scrambled condition because the location of the fragment was kept constant between and experts could have surmised a fragment’s location based on where it is usually located in a typical fingerprint. For example, if they know that deltas tend to appear in the bottom corners of a fingerprint, then they may also appear in the same location in a scrambled image. Alternatively, an expert’s visual search skills may be somewhat domain-general such that they are able to efficiently search for features even among novel stimuli. It is unclear, however, why a domain-general visual search ability would not also include an advantage searching for less useful features.

A more reasonable explanation for why experts performed well when locating useful fragments within scrambled prints is a superior working memory for useful features. During visual search, one must hold in mind the target feature and then find it in the array. Maturi and Sheridan (2020) have shown that when searching for a section of a musical score within a larger array, experts returned to the target feature far less than novices. Switching between target and array less frequently suggests that experts have a richer working memory for the target feature than novices. Similarly, experts in our study may have been more efficient than novices when searching for useful features even in scrambled fingerprints because they could hold more of the featural information in mind during the task. Perhaps efficiently searching for features depends both on the context of the target and a superior visual working memory for the target itself, and these two effects may be additive.

## Experiment 2

In Experiment 1, we investigated how well domain experts and novices could locate points of correspondence, whereas in Experiment 2, we investigate their ability to locate points of difference using the 'Spot-the-Difference' task. In this task, participants are instructed to spot differences between two otherwise identical images. While the Find-the-Fragment task is in many ways an adaptation of traditional visual search tasks, the Spot-the-Difference task resembles a comparative visual search task or static change detection task. Mirroring Experiment 1, we manipulate whether the target is more useful or less useful and whether participants search between intact prints or scrambled prints.

When comparing a latent fingerprint to a rolled print, examiners must seek out not only corresponding features, but also any features that are different. The side-by-side comparison inherent to the Spot-the-Difference task is therefore quite similar to what fingerprint examiners routinely encounter in their everyday workflow. Moreover, processing alternative solutions or evaluating disconfirmatory evidence can aid problem solving more generally. For example, toggling back and forth between two misaligned images can improve a mammographer’s diagnostic accuracy (Drew et al., 2015). Detecting differences between prints may therefore be a distinct but critical skill that examiners possess.

As discussed in the introduction to this paper, perceptual experts are highly sensitive to the most useful domain-specific information, and their perceptual skills hinge on domain structure. Studies from change detection paradigms support these findings further. For instance, football players are more efficient than novices at detecting meaningful changes to football scenes (e.g., the ball being removed), but not less meaningful changes (e.g., a change to the referee’s glove) or changes to scenes unrelated to football (e.g., traffic scenes; Werner & Thies, 2000). People are also better at identifying features and detecting changes to upright faces—an orientation that is familiar—far more readily than inverted or scrambled faces (Buttle & Raymond, 2003; Tanaka & Farah, 1993). We therefore anticipate a three-way interaction just as we did in Experiment 1. We expect fingerprint examiners to outperform novices, but only at spotting differences between useful features located in intact fingerprints, and we predict that the group difference in this condition will be large.

## Method

### Preregistration

All methods, materials, event sequences, experimental code, sensitivity analyses, hypotheses and planned analyses were preregistered on the Open Science Framework prior to collecting data. The preregistered project can be found here: https://osf.io/aphxg

### Participants

All 88 participants in Experiment 2 (44 experts and 44 novices) were the same as in Experiment 1 because each participant completed both tasks in the same testing session. Novices were compensated in the same manner as in Experiment 1.

### Design, procedure and measures

**Fig. 4:**
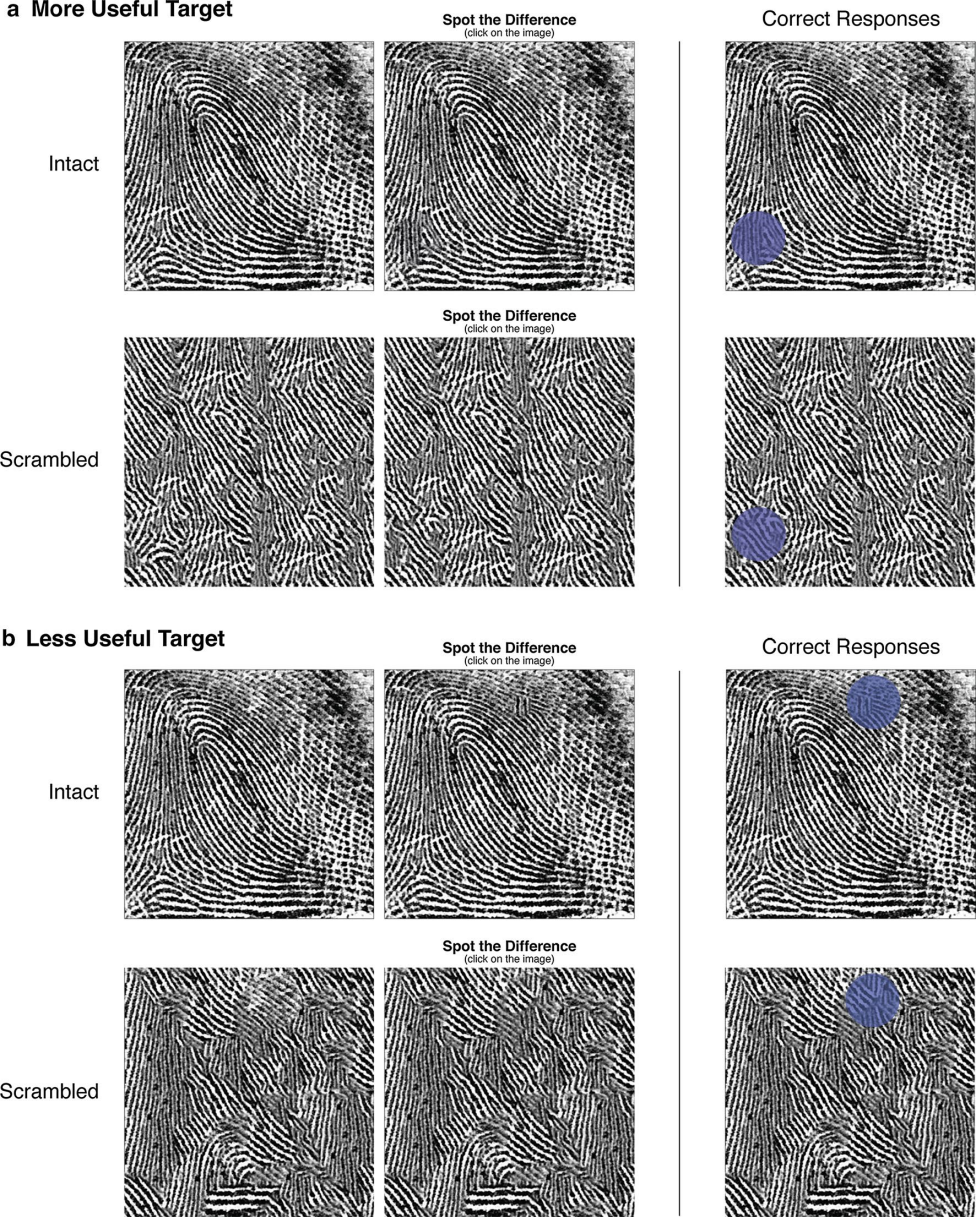
A schematic diagram of the Spot-the-Difference task used in Experiment 2. We provide an example of each of the trial types on the left: either two intact fingerprints were displayed or two scrambled prints, and the target change had been made to either a ‘more useful’ feature (**a**) or a ‘less useful’ feature (**b**). When generating the scrambled fingerprints, we kept the location of the target change identical to those in the intact prints. The correct location of the change in each example is displayed on the right

The design, procedure and software used in Experiment 2 were identical to Experiment 1 except for the nature of the task (see Fig. 4). On each of the 48 trials, participants were presented with two identical fingerprint images side-by-side, except for one change in the image on the right—a circular section we swapped out for alternative ridge information. Participants were instructed to spot this difference as quickly as they could. On half of the trials, the two images presented were two intact fingerprints, and in the other half, two scrambled fingerprints were presented. In addition, half the trials included a change made to a more useful feature and in the other half of the trials the change had been made to a less useful feature. We kept the location of these changes equivalent when creating the scrambled images. For each trial, we measured accuracy and response time. We also use BIS as our measure of speed–accuracy for the same reasons outlined in Experiment 1, but all data analytic scripts for each measure can be found here: https://osf.io/my28c.

### Materials

*Intact images* The 72 intact fingerprint images (750 × 750 pixels) in this task were identical to the intact fingerprints from Experiment 1. However, we also created altered versions of each print where we made a change to some feature. We used the *more useful* and *less useful* points gathered by Robson et al. (2020) as center points to generate circular changes of different sizes in each of the 72 intact prints. In each case, we deleted the selected area and replaced it with alternative information using Photoshop’s Content Aware Fill tool. In a pilot study (https://osf.io/3g4e7), we gave 48 novices a Spot-the-Difference task where the size of the change gradually increased every five seconds. These novices could spot the change about 50% of the time when it was 3.71% the size of the image that it was located in. We used changes of this size as it was equidistant from floor and ceiling. For each of the 72 fingerprints, we created a pool of 16 altered images; eight had a change made to a more useful feature and eight had a change made to a less useful feature.

*Scrambled images* We generated a scrambled image for each of the 16 variants of every intact fingerprint. We scrambled them in the same way as we did in Experiment 1, except the area we kept unchanged was larger this time (3.71% the size of the image). In total, we generated 1152 scrambled prints (750 × 750 pixels). For each of the scrambled images, we also created an altered variant just as we did for each intact print.

## Results

**Fig. 5:**
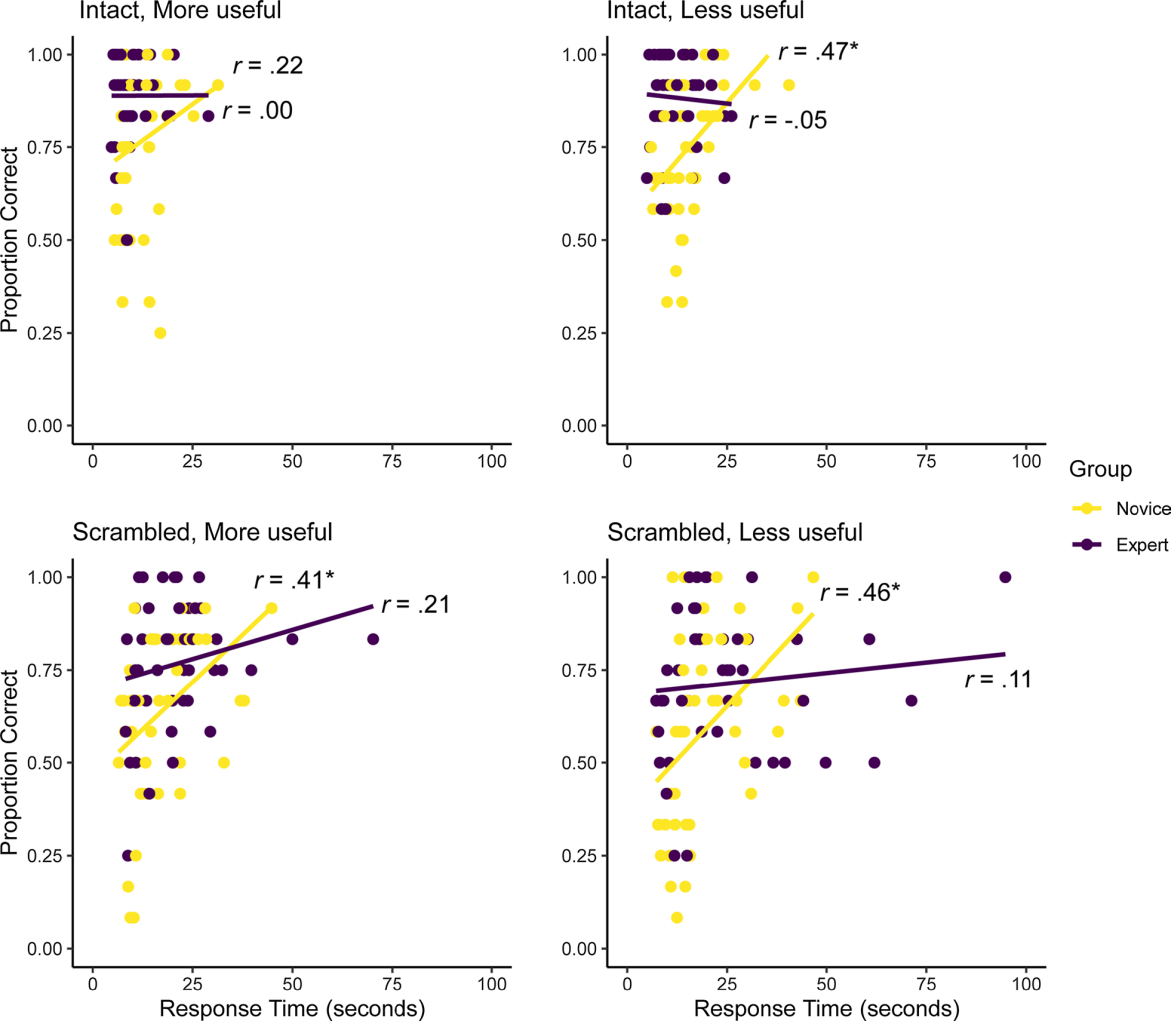
The correlations between speed and accuracy for each group across the four conditions in the Spot-the-Difference task. An asterisk (*) signifies a significant correlation (*p* < .05)

We found that participants who took more time to make a decision in the Spot-the-Difference task tended to be more accurate, *r*(86) = 0.50, *p* < 0.001. Among novices, accuracy and response time were positively correlated, *r*(42) = 0.68, *p* < 0.001, as well as among experts, *r*(42) = 0.36, *p* = 0.015. The speed–accuracy correlations for each condition are presented in Fig. 5. We only report on speed–accuracy here as it appears more suitable than accuracy as a measure of performance.

### Speed–accuracy (BIS)

**Fig. 6:**
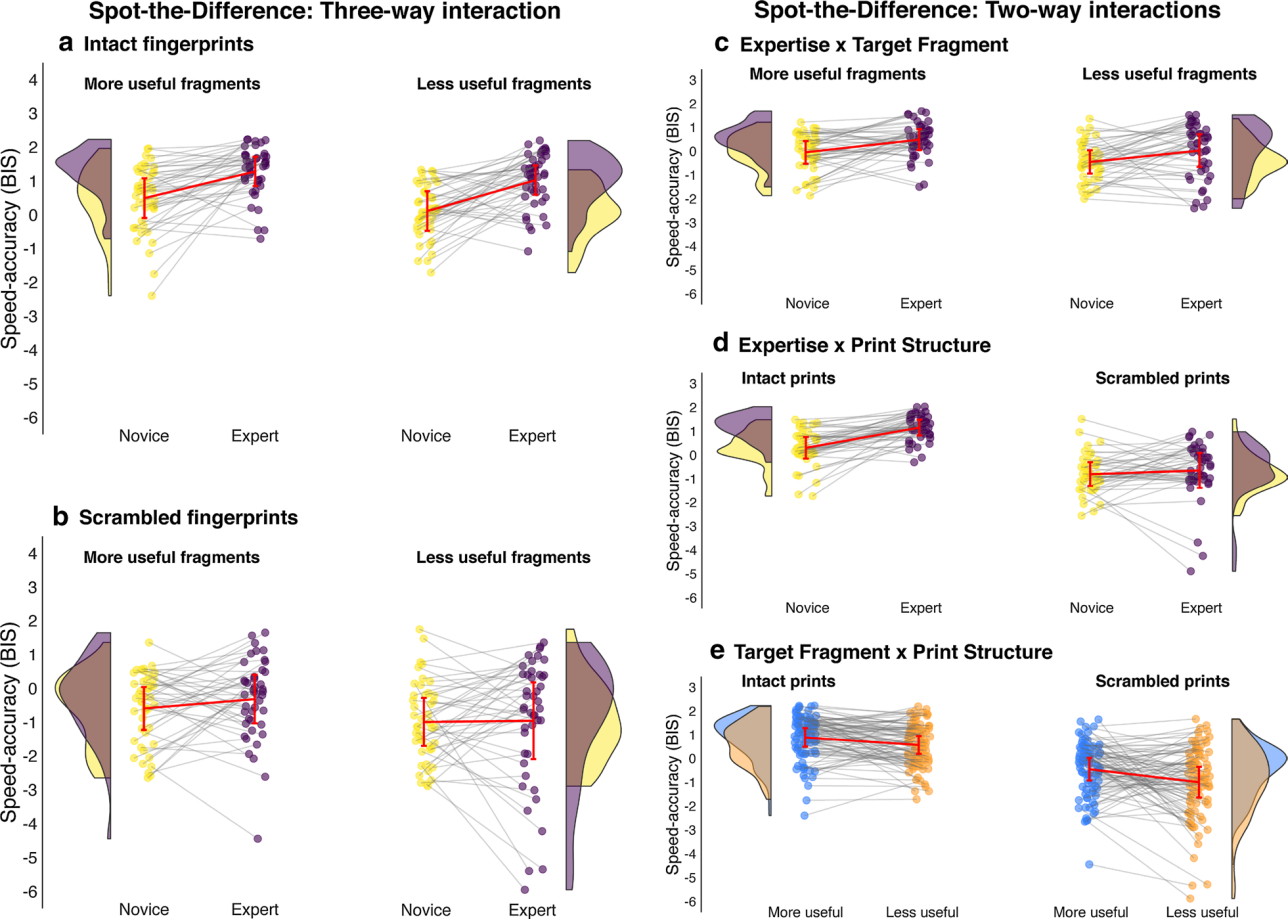
A visualization of the interactions for the speed–accuracy data (measured using Balanced Integration Scores) in the Spot-the-Difference task. We present the three-way interaction (left) comparing experts and novices for each type of change for the intact trials (**a**) and scrambled trials (**b**). We also present all three two-way interactions (**c**–**e**). In all but panel **e**, the expert data are colored purple and the novice data yellow. Each participant’s individual performance is represented by the small circles, with grey lines connecting every expert to their yoked novice ‘twin’. The distributions depict the overlap in each group’s performance, the red error bars represent the confidence interval around each cell mean, and a red line connects the group means for each cell. **e** Depicts the final two-way interaction comparing performance at finding more useful changes (blue) and less useful changes (orange) overall with each participant’s data connected by a grey line

We performed a parametric test (three-way mixed methods ANOVA) and a nonparametric test (three-way mixed ART factorial analysis) on the BIS data. The pattern of effects was identical in both models, so we only report the parametric ANOVA here (see Fig. 6). Counter to our predictions, the 2 (Expertise) × 2 (Target) × 2 (Structure) mixed ANOVA revealed no significant three-way interaction, *F*(1, 86) = 0.912, *p* = 0.342. The Expertise × Target interaction was nonsignificant, *F*(1, 86) = 0.07, *p* = 0.790, and the Target × Structure interaction was also nonsignificant, *F*(1, 86) = 1.41, *p* = 0.239. However, there was a significant Expertise × Structure interaction, *F*(1, 86) = 10.04, *p* = 0.002, *η*^2^_G_ = 0.025. Experts (*M* = 1.15, SD = 0.75) were better than novices (*M* = 0.3, SD = 0.89) at spotting differences between intact fingerprints *F*(1, 174) = 49.98, *p* < 0.001, *η*^2^_G_ = 0.213, whereas experts (*M* = − 0.65, SD = 1.57) and novices (*M* = − 0.81, SD = 1.11) performed similarly at spotting differences between scrambled prints, *F*(1, 174) = 0.59, *p* = 0.445.

### Exploratory analyses

*Simple interactions* We explored why we did not find the expected three-way interaction by observing simple interactions. We expected experts to outperform novices only when spotting changes to useful features in intact prints, but we found no significant Expertise × Target interaction for the intact trials, *F*(1, 86) = 0.35, *p* = 0.559. Experts (*M* = 1.28, SD = 0.71) were indeed better than novices (*M* = 0.49, SD = 0.96) at locating changes to more useful features in intact prints, *F*(1, 86) = 19.16, *p* < 0.001, and the effect was large (*η*^2^_G_ = 0.182). However, experts (*M* = 1.02, SD = 0.78) were surprisingly better than novices (*M* = 0.11, SD = 0.77) at locating changes to less useful features in intact fingerprints as well, *F*(1, 86) = 30.43, *p* < 0.001, and the effect was larger still (*η*^2^_G_ = 0.261). As expected, we found no significant Expertise × Target interaction when observing only the scrambled trials, *F*(1, 86) = 0.45, *p* = 0.506, Experts (*M* = − 0.32, SD = 1.18) and novices (*M* = − 0.59, SD = 1.05) did not differ at spotting changes to more useful features in scrambled prints, *F*(1, 86) = 1.36, *p* = 0.247, nor did experts (*M* = − 0.98, SD = 1.13) and novices (*M* = − 1.02, SD = 1.15) differ at locating changes to less useful features in scrambled prints, *F*(1, 86) = 0.12, *p* = 0.912.

*Correlating performance and experience* We found no significant correlations between performance on the Spot-the-Difference task and years of experience as an examiner. Years of experience did not correlate with accuracy in the task, *r*(42) = − 0.25, *p* = 0.103, nor did years of experience and speed–accuracy, *r*(42) = − 0.27, *p* = 0.080. There was also no correlation between years of experience and accuracy in the useful-intact condition, *r*(42) = − 0.19, *p* = 0.205, nor years of experience and speed–accuracy for this condition, *r*(42) = − 0.28, *p* = 0.062.

## Discussion

In Experiment 2, we set out to compare fingerprint examiners and novices on a fingerprint comparative visual search task. We also sought to understand how the usefulness of the target and the structure of the array affect performance. We expected that experts would outperform novices only at spotting useful changes to intact fingerprints, but not in any other condition. We did not find the expected three-way interaction, but our results partially support our hypothesis. Experts were indeed better than novices at locating differences between intact fingerprints, but not scrambled prints. Their visual search abilities therefore do not appear to generalize beyond the stimuli that they have been exposed to in their formal training and everyday workflow. This finding lends support to a domain-specific account of perceptual expertise (Carrigan et al., 2019; Chase & Simon, 1973a, b; Curby et al., 2009; Diamond & Carey, 1986, 2009; Sims & Mayer, 2002) given that the experts’ perceptual advantages dissipated when the familiar domain structure was scrambled.

Contrary to our expectations, experts and novices performed similarly not only when spotting changes to useful features, but also when spotting changes to less useful features. Because perceptual experts in many domains are more sensitive to the most useful features of their domain (Krupinski, 1996; Krupinski et al., 2013; Mann et al., 2007; Maturi & Sheridan, 2020; Reingold et al., 2001; van der Gijp et al., 2017; Ziv, 2016), we predicted that examiners would be more efficient than novices at spotting changes to highly relevant areas, but not to less relevant areas. This was not the case. However, the nature of the task may have forced examiners to approach the task differently to how they might approach their routine work. In reality, it is likely that examiners seek out clusters of features in one print and then see whether this cluster is also present or absent in a comparison print. In this scenario, examiners may pick out the set of features that will be most helpful for confirming or disconfirming whether the prints match. However, participants in the Spot-the-Difference task are not cued as to whether a change will be located in a useful or less useful region. Without this information, it would be counterproductive to hedge one’s bets on the regions and features that one might be most sensitive to because the change could just as likely be in a less useful region. Instead, the examiners may have outperformed novices in the intact trials regardless of the location of the change because experts, as demonstrated in several domains (e.g., Carrigan et al., 2019; Chase & Simon, 1973a, b), tend to process domain-relevant stimuli more efficiently than novices.

## Comparing performance across the two tasks

We found a moderate positive correlation between speed–accuracy in the Find-the-Fragment task and speed–accuracy in the Spot-the-Difference task, *r*(86) = 0.50, *p* < 0.001. The correlations were similar among novices, *r*(42) = 0.48, *p* = 0.001, and among experts, *r*(42) = 0.44, *p* = 0.003. Overall, the relationship was weaker but still positive in the useful-intact condition, *r*(86) = 0.30, *p* = 0.005. However, the relationship between speed–accuracy on each task for this condition was nonsignificant among experts, *r*(42) = − 0.07, *p* = 0.640, and nonsignificant among novices, *r*(42) = 0.20, *p* = 0.19.

We also conducted a binomial logistic regression to establish whether performance in the useful-intact variations of each task could correctly classify participants as either an expert or a novice. Speed–accuracy on the useful-intact versions of each task predicted expertise, χ^2^ (2, *N* = 88) = 36.72, *p* < 0.001, together explaining 30.1% of the variance. In fact, performance on both tasks contributed to the model. Every increase of one in BIS on the Find-the-Fragment task translated to 3.15 (*b* = 1.15, *p* < 0.001) times greater odds of being an expert, and every increase of one in the BIS on the Spot-the-Difference task translated to 3.32 (*b* = 1.20, *p* = 0.002) times greater odds of being an expert. A dominance analysis revealed that Find-the-Fragment performance was the most important predictor, contributing an average of 17.5% of the variance, whereas Spot-the-Difference performance contributed an average of 12.6%.

## General discussion

Across two experiments—one where participants searched for points of correspondence and one where they searched for differences—we tested whether domain experts have superior visual search skills than novices. We also examined whether any visual search advantages are constrained to useful target features and to images containing intact domain structure. In both experiments, we hypothesized that fingerprint examiners would be better than novices at spotting useful features and changes (but not less useful features and changes) in intact fingerprints (but not scrambled prints). We did not find the expected three-way interaction in either experiment, but our findings nonetheless supported our hypotheses to a large extent. Across both experiments, experts were generally more efficient at searching for targets compared to novices when these targets were located in the intact fingerprints, but not when they were located in the scrambled prints. Just as a chess expert’s recall for chess pieces is disrupted when the pieces are randomly arranged (Chase & Simon, 1973a, b; Gobet & Simon, 1996), the fingerprint examiners demonstrated more efficient search when faced with intact fingerprints but performed like novices when the prints were scrambled. This finding suggests that disrupting the visual structure of a fingerprint (e.g., by scrambling the image) removes the usual structural cues surrounding target features, effectively disabling expert search patterns. This visual context specificity is in line with the existing body of evidence in perceptual expertise in other domains (e.g., Carrigan et al., 2019; Curby et al., 2009; Diamond & Carey, 1986, 2009; Sims & Mayer, 2002). An expert’s visual search ability appears to depend on experience with the structural regularities of that domain.

In Experiment 1, the fingerprint examiners also appeared to be highly sensitive to useful domain features, but not to less useful features. They were far more efficient than novices at locating useful features, but equal to novices at locating less useful features. Perceptual experts across a range of domains demonstrate a similar sensitivity to the most useful features and regions for the task at hand (e.g., Krupinski, 1996; Mann et al., 2007; Maturi & Sheridan, 2020; Reingold et al., 2001; Ziv, 2016). The years of experience that experts have accumulated with various cues and response options enables them to learn which features are the most relevant to the task (e.g., Lansdale et al., 2010; Sturman et al., 2019). This fine-tuning to cue-based associations can enhance the saliency of useful features and draws an expert’s attention.

In sum, our results suggest that professional fingerprint examiners possess superior visual search skills compared to novices. However, their skills are limited to domain-specific stimuli and to features that are most relevant to their routine decisions. Our results say little about the proficiency of these examiners, nor how often they might make mistakes, but they do speak to the nature of their expertise. The fact that an examiner’s visual search ability is highly constrained by the stimuli used in the task is a finding that we would expect from a group that possesses genuine expertise. Nevertheless, our results deviated somewhat from our expectations. Below we discuss why this might be, along with the broader implications of our findings.

Experts in the Find-the-Fragment task outperformed novices at finding useful fragments in both intact and scrambled fingerprints. Therefore, an expert’s sensitivity to useful features may aid their visual search even when a target is situated in an unfamiliar context. Experts possess a rich visual working memory for useful features, which means they need to switch between target and array far less than a novice would (e.g., Maturi & Sheridan, 2020). Superior visual working memory therefore appears to facilitate search in addition to, and largely independent of, the advantages that contextual cueing provides. We also found that experts were better than novices at spotting both more useful and less useful changes in the Spot-the-Difference task when these changes were situated in intact prints. Unlike the Find-the-Fragment task, participants had no cue as to what needed to be located and therefore where the change may be located. Examiners likely outperformed novices because, like many experts, they process domain-specific stimuli more efficiently than novices (Carrigan et al., 2019; Chase & Simon, 1973a, b; Maturi & Sheridan, 2020). A superior visual working memory and the nature of the task can therefore boost or diminish an expert’s visual search ability, and this may explain why our results did not align entirely with our original hypotheses.

Despite the differences between the two tasks, performance on one task correlated with performance on the other. This association suggests that the tasks tap into a similar underlying ability, presumably visual search efficiency. However, in the only condition where we expected an expert-novice difference—the useful intact condition—the relationship between the two tasks was weaker. We also found that speed–accuracy performance for the useful-intact variants of both tasks uniquely contributed to predicting expertise, together explaining almost one-third of the variance. Efficiently locating corresponding features and locating differences may be two unique and necessary skills to reach expert levels of performance in a domain. However, many other perceptual abilities underlie feature comparison expertise, including working memory and statistical learning (see Growns & Martire, 2020).

Our results also revealed that neither the Find-the-Fragment task nor the Spot-the-Difference task could distinguish between examiners with more experience from those with less. Perhaps the tasks were not sufficiently sensitive to parse the varied levels of skill within our sample of experts. Alternatively, self-reported years of experience is a poor measure of actual domain expertise because it can vary considerably from one person to the next and does not capture the quality of one’s training (Carrigan et al., 2020). There is also evidence that competence in a domain plateaus within a few years (Choudhry et al., 2005; Ericsson, 2004). The skills of fingerprint trainees, for instance, seem to reach asymptote within a year of their traineeship (Searston & Tangen, 2017a). In future studies, superior measures of experience (Sunday et al., 2018) and more valid measures of job performance are needed to test these claims.

In any case, our findings have implications for recruitment and training. Some police agencies in the past have recruited forensic pattern matchers, such as fingerprint examiners, using tasks that supposedly measure general visual comparison abilities. It may be that general skills before training predict who flourishes later on, but there is no evidence that this is true for fingerprint identification. Moreover, if an ability to search for features only in domain-specific stimuli is what separates experts from novices, as we demonstrate here, then time and resources may be wasted by recruiting individuals with tasks that are unrelated to the domain.

Learning to efficiently locate features and spot differences between prints may also help one develop the ability to match fingerprints. Training programs of this kind, such as attentional highlighting, are already being tested (Roads et al., 2016), but it is still unclear whether this training actually improves matching ability. The Find-the-Fragment and Spot-the-Difference tasks, at least in their useful-intact forms, may prove to be useful training tasks for developing pattern matching expertise. Nonetheless, both tasks ought to be adapted to reflect the complexity of the decisions that examiners typically make. During their day-to-day work, examiners typically compare fingerprint impressions that appear very different from one another. However, in the Find-the-Fragment task, the fragment appears exactly (pixel for pixel) as it does in the array, and the two images in the Spot-the-Difference task are almost entirely identical, except for the change that needed detecting. An understanding of the variation within and between identities is crucial to many forms of perceptual expertise (Kramer et al., 2018) and an effective training program ought to instill this appreciation.

## Conclusions

This is the first study to investigate how fingerprint examiners and novices differ in their visual search ability, and how the target feature and structure of the stimuli affect performance. Across both tasks, examiners demonstrated more efficient search than novices, but only for images specific to their domain of expertise. Although these experts performed exceptionally well at locating features in typical fingerprints, scrambling the structure of these prints reduced expert search performance to novice levels. In our first experiment, examiners also efficiently located ‘useful’ features that they typically encounter in their routine decisions. However, their performance fell to novice levels when locating less useful features. Visual search ability in the domain of fingerprint identification clearly hinges on domain-specific experience and on the attentional fine-tuning that results from extensive training.

## Data Availability

The data for each novice and expert participant, and the code used to produce our results and plots, are available on the Open Science Framework, with the exception of identifiable demographic information (Experiment 1: https://osf.io/azesx Experiment 2: https://osf.io/aphxg). The images, experimental software, event sequences can also be found through these links.

## Abbreviations

RCS: Rate Correct Score
BIS: Balanced Integration Score
ART: Aligned Rank Transform

## Footnotes

1 Content Aware uses an algorithm called PatchMatch to approximate nearest neighbor matches between image patches (small, square regions; Barnes et al., 2009).
